# Exosomes: A Promising Cell-Free Therapeutic Tool for Treating Cutaneous Nerve Injuries and Promoting Wound Healing

**DOI:** 10.3390/ijms26115323

**Published:** 2025-06-01

**Authors:** Yujie Mu, Ruting Luo, Le Zhao, Danting Chen, Lixin Cao, Zhenkai Jin, Kun Li, Min Wang

**Affiliations:** 1 School of Light Industry Science and Engineering, Beijing Technology and Business University, Beijing 102401, China; muyujie5576@163.com (Y.M.); lrt18948322588@163.com (R.L.); 18873262945@163.com (D.C.); c1258925380@163.com (L.C.); 2 School of Pharmacy, Henan University of Chinese Medicine, Zhengzhou 410198, China; zhaole@hactcm.edu.cn; 3 BTBU-YHZK Bioengineering Raw Material Development Laboratory, Beijing Technology and Business University, Beijing 102401, China; jinzhen_kai852@126.com (Z.J.); likun_135@126.com (K.L.)

**Keywords:** exosome, nerve, nerve regeneration, wound, wound healing

## Abstract

The skin is the body’s largest organ. It serves various functions, including protection and metabolism. Due to its structure and location, it is more vulnerable to external physical and chemical damage than internal organs. Additionally, certain endogenous diseases can cause pathological changes to appear on the skin and nerves. When skin tissue breaks down or sustains severe trauma, the cells, blood vessels, and nerves across all layers can suffer varying degrees of damage. This often results in pain, itching, sensory disturbances, and other discomforts, causing significant distress to patients. Stem-cell-derived exosome therapy has emerged as a promising treatment for skin injuries due to its safety, non-toxicity, and precision medicine benefits. Research has shown that stem-cell-derived exosomes regulate nerve cells by mediating MicroRNA (miRNA) transport and expression between cells, promoting axon growth. This exosome-driven miRNA exchange serves as a vital mode of intercellular communication, playing a crucial role in nervous system repair. Nerves play a critical role in skin wound healing and tissue regeneration, with sensory and autonomic nerves influencing key skin functions such as inflammation, immune defense, apoptosis, proliferation, and wound repair. Exosomes may aid in treating cutaneous nerve injuries by directly or indirectly promoting axon regeneration, nerve cell proliferation, and the release of protective neurofactors.

## 1. Introduction

**Figure 1:**
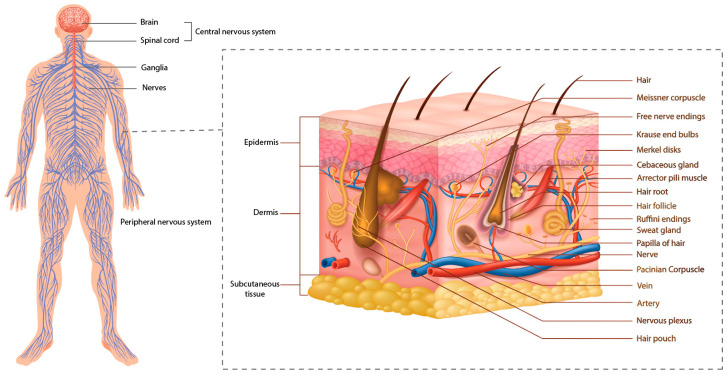
Cross-section outlining the distribution of the cutaneous nervous system in the skin.

The skin, a unique organ covering the body, is susceptible to the external environment and responds actively to trauma and endogenous diseases [1]. These functions rely on the neural network within the skin tissue (Figure 1). Severe damage to skin tissue reaching the reticular layer, such as deep burns, does not regenerate and results in scarring after healing. In contrast, the papillary and epidermal layer damage can undergo regenerative repair. This repair and scar formation process is highly complex and regulated by various cells, the extracellular matrix, cytokines, and neuroimmune mechanisms [2]. Nerves nourish and regulate the skin, which is crucial to wound healing. During this process, the inflammatory response activates sensory and motor nerves in the dermis, prompting the release of neuropeptides that regulate healing [3]. Skin regeneration, repair, and wound healing are closely tied to nerve function, highlighting the nerve’s essential role in these processes.

The skin is a highly sensitive organ that relies on nerves for proper operation. It forms a neuroimmunoendocrine system by connecting with the peripheral sensory, autonomic, and central nervous systems. Skin cells can secrete neurotransmitters, hormones, and inflammatory mediators, operating within a regulated neuro–endocrine–immune network that functions through precise feedback loops [4] (Figure 1). The skin and nervous system are closely connected, with all areas of the skin containing specific structural components, cells, and immune cells. One key function is detecting and transmitting exogenous and endogenous danger signals to immune cells, triggering a coordinated immune response. When exposed to external stimuli, the body’s neuro–endocrine–immune network quickly adjusts cellular metabolism, neural excitation, and hormone release while initiating gene regulation to trigger various biological responses [5]. In skin trauma repair and regulation, nerve factors are as important as the regenerative effects of blood vessels and tissues. Tissue damage or ischemia often accompanies nerve damage, and nerve regeneration occurs alongside wound repair, playing a crucial role in the overall healing process.

Skin nerve injuries typically cause pain, itching, and sensory disturbances and can result in lifelong disability, significantly impacting patients’ quality of life [4]. Recently, tissue-engineered transplants with cells and neurotrophic factors have been developed for autologous nerve transplantation. However, due to the complexity of the nervous system, this approach still faces challenges in achieving optimal clinical outcomes. Neural regeneration after organ and tissue injury remains a significant hurdle for clinicians and researchers.

**Figure 2:**
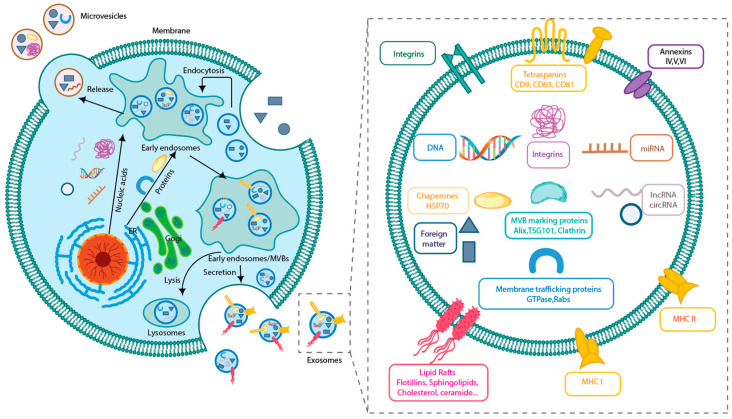
Exosome production process and structure. Exosomes carry information from parent cells and play a key role in information transfer.

Exosomes are extracellular membrane nanovesicles secreted by most cells, and they play a key role in intercellular communication (Figure 2). Exosomes carry a variety of biologically active molecules, including proteins, nucleic acids, lipids, and metabolites. These components are characterized by selective packaging that reflects the molecular characteristics of their cell of origin [6,7]. Exosomes bind to receptor cells via surface proteins or lipids or by directly fusing with cell membranes through endocytosis. This delivers signaling molecules (e.g., microRNAs and proteins) and regulates the gene expression, proliferation, and migration of target cells. The double membrane structure of exosomes protects their contents (e.g., RNA) from degradation and stabilizes them in body fluids [8]. In the nervous system, they are involved in both physiological and pathological processes. Exosomes promote wound healing and inhibit scar formation by regulating inflammation, cell proliferation and migration, angiogenesis, and collagen deposition during skin wound healing. Additionally, they support neurotherapeutic functions by mediating axon regeneration, Schwann cell activation, vascular regeneration, and inflammation regulation, thereby aiding in the repair of neurological deficits and improving the quantity and quality of nerve fibers. This contributes positively to the repair of skin nerves [9,10]. Compared with traditional surgical transplantation and drug therapy, stem cell exosomes offer similar therapeutic effects and functional properties as their parent cells while avoiding the ethical and rejection issues associated with embryonic stem cell transplantation. Thus, developing stem cell exosome therapy for cutaneous nerve injury is of high clinical value.

This paper systematically reviews the causes of cutaneous nerve injury, the relationship between nerve and skin repair, the mechanism of stem cell exosomes in treating cutaneous nerve injury, and their research progress. It also discusses the future challenges and potential clinical applications of stem cell exosomes.

## 2. Cutaneous Nerve Injury

As the body’s primary barrier, the skin is highly exposed to external physical and chemical factors and chronic diseases, which can cause damage. This paper focuses on damage to the skin’s nervous system, the regulation of its neuro–endocrine–immune function, and the various factors contributing to this damage. Skin tissue regeneration and repair is a complex, time- and space-dependent process involving multiple cells, and it is regulated by different factors. Repairing the nervous system within and near the skin is especially intricate following damage.

### 2.1. Causes of Cutaneous Nerve Injury

Most peripheral nerve injuries result from direct mechanical trauma to specific nerves, particularly those located in superficial anatomical pathways or regions prone to external impact. Such injuries compromise the structural integrity of neural tissues, ultimately resulting in functional impairments in neural signal conduction. Skin nerves can suffer varying degrees of damage from external factors like UV radiation, burns, surgical wounds, and severe impacts affecting the nervous system within the skin. Endogenous factors can also contribute to nerve damage. Conditions such as diabetes, endocrine and immune dysfunctions, leprosy, psoriasis, and other chronic diseases can lead to nerve damage, severely affecting the patient’s quality of life.

#### 2.1.1. External Factors

**Ultraviolet ray**

Solar UV radiation can damage the skin’s integrity, leading to chronic inflammation, aging, and cancer. It penetrates the epidermis and induces various biological effects in the skin’s nervous system [11]. The dermis connects to the central nervous system (CNS) through sympathetic and sensory peripheral nerves. Sensory innervation originates from neurons in the dorsal root ganglia, while peripheral fibers of postganglionic sympathetic neurons provide sympathetic innervation. The free nerve endings of these fibers extend into the epidermis, where they closely interact with keratin-forming cells, melanocytes, and Langerhans cells [12]. UV irradiation reduces the number of epidermal and calcitonin gene-related peptide-positive dermal nerve fibers while also suppressing the immune response. This helps relieve itching and reduce the severity of conditions such as rashes, eczema, and hives [13]. Rodriguez et al. [14] found that UVA and UVB reduced the density of nerve endings by 40–60% in the control and placebo groups. This modest reduction in skin nerve fiber density may contribute to the therapeutic effects of UV irradiation in reducing itching in conditions such as psoriasis and atopic dermatitis. However, prolonged UV exposure can lead to erythema, ROS production, DNA damage, increased P53 production, and apoptosis. This results in skin photoaging, and cumulative UV-related damage may contribute to the development of skin cancer [15]. Data from Fonseca et al. [16] showed a significant reduction in cutaneous nerve fibers following UV irradiation and decreased skin cells with peripheral free nerve endings. This leads to the phenomenon of cutaneous nerve damage and cell apoptosis. An appropriate UV irradiation intensity can help relieve skin inflammation, but prolonged exposure can cause skin damage and lesions. UV radiation activates subcutaneous inflammatory cytokines and alters TGF-β expression, which regulates extracellular matrix remodeling. This, in turn, affects the proliferative activity of subcutaneous neural cells, ultimately leading to skin and cutaneous nerve damage.

**Burns**

A burn is an injury to skin tissue caused by heat, radiation, radioactivity, electricity, friction, or contact with chemicals [17,18]. Burns can be categorized into mild, moderate, and severe burns. Mild burns mainly damage the epidermis and part of the dermis, which may result in direct thermal damage to local nerve endings or pain caused by the release of inflammatory mediators [19]. Moderate burns involve the deeper layers of the dermis, destroying more nerve endings and peripheral nerve branches, resulting in pain with partial sensory loss. Such burns may exacerbate intraneural scarring due to thermal injury or secondary inflammation, which affects nerve regeneration [20]. Severe burns, also known as total burns, are total burns that destroy the skin, subcutaneous tissue, and peripheral nerve structures, resulting in motor and sensory dysfunction. Electrical and chemical burns are often accompanied by irreversible neural axonal damage, leading to chronic peripheral neuropathy [21,22]. Beyond restoring sensory perception, nerve fibers are vital in skin repair and in maintaining dynamic skin homeostasis during the wound-healing process [23]. Autologous skin grafting is the most effective treatment for cutaneous burns but is limited in patients with insufficient donor skin due to extensive injuries. Additionally, peripheral nervous system damage can impair wound healing, causing delays or chronic wounds. This highlights the critical role of nerves and neuromodulators in skin tissue repair. Tissue engineering treatments, including biomaterials, autologous skin grafts, skin substitutes, and stem cell technology, offer promising alternatives for wound healing, potentially enhancing sensory recovery and improving patient outcomes [24,25,26].

**Electroshock**

Neurological complications from electrical injuries vary widely and may appear immediately or up to 2 years after the incident. These injuries are classified as high voltage (over 1000 V) or low voltage (under 1000 V). Most electrical injuries result from low-voltage sources, while high-voltage injuries are typically associated with industrial accidents or electrical line work. Electrical injuries are classified into four levels. First-degree wounds are superficial, characterized by reddening, and usually do not require surgical intervention. They are often managed with topical moisturizers to prevent re-injury. Second-degree wounds involve superficial edema, affecting the interface between deeper living tissue and the superficially injured tissue. The surface may appear moist, with varying degrees of blistering and rupture, while the deeper skin layers remain intact, enabling epithelial regeneration. Third-degree wounds are deeper injuries characterized by whitened, darkened, or dry, leathery skin. Wounds larger than 2 cm usually require surgical debridement and skin grafting. “Grade IV wounds” refer to extensive damage involving deeper soft tissues, such as subcutaneous fat, muscle, or bone [27]. High-voltage electric shocks often cause multiple skin burns and significant internal thermal injuries. Neurological damage is also typical, ranging from mild to severe, and may include cognitive impairment and sensory abnormalities.

**Slam**

Severe impacts or repetitive training can significantly damage the skin’s nervous system. Air crash survivors often present with peeled skin, sharp edges, and hemorrhaging beneath the articular cartilage. However, peripheral nerves are typically better preserved due to their greater resistance to tearing [28]. Strenuous sports like volleyball, where athletes repeatedly serve and spike the ball with force, can cause injuries to the right median palmar cutaneous nerve due to repetitive trauma to the forearm and wrist [29]. Prolonged archery training increases the risk of acute upper extremity injuries, including finger nerve and arterial lacerations, forearm contusions, and finger nerve compression from the bowstring. Chronic injuries may include bilateral medial epicondylitis, median nerve compression at the wrist and elbow, and De Quervain’s tenosynovitis [30]. Damage to the skin’s nervous system from violent impacts and high-intensity repetitive training is cumulative and often takes longer to manifest compared to mechanical injuries.

#### 2.1.2. Endogenous Factors

**Figure 3:**
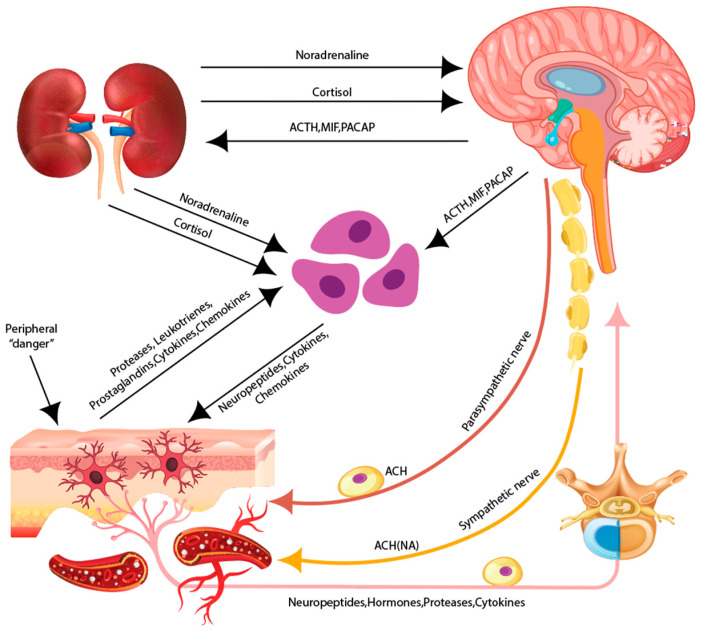
The skin as a neuroimmunoendocrine organ. The skin is associated with the peripheral sensory nervous system (PNS), the autonomous nervous system (ANS), and the central nervous system (CNS). Various stressors activate the hypothalamus/hypophysisis within the CNS which results in the release of neuromediators.

Like the nervous system, the skin originates from the ectoderm, making its nerve fibers and specialized mechanoreceptors as complex as those in the CNS. Along these fibers are immune cells, mast cells, dendritic cells, macrophages, and intrinsic lymphocytes. The skin communicates with these cells through neurotrophins and neuropeptides released by nerves, known as “neuro-immune interaction” (Figure 3).

The skin functions as a target organ for various hormones and an endocrine organ, with roles comparable to the hypothalamic–pituitary–adrenal (HPA) axis. It has receptors for glucocorticoids, thyroid hormones, insulin, and insulin-like growth factor 1 (IGF-1). Additionally, it expresses glucocorticoid-synthesizing enzymes, including CYP11A1, CYP17A1, and CYP11B1, enabling glucocorticoid synthesis [31]. The skin functions both as a target for endocrine hormones and as a site for their production [32]. The skin communicates through nerves and receptors, with its nervous system comprising skin cells and nerve endings that release various neurotransmitters. Endocrinologically, skin tissue acts as a target for chemical messengers, while its hormones regulate functional stability, maintaining homeostasis [33]. From an immunological perspective, the skin’s immune system comprises humoral and cellular immunity, with immune cells primarily located in the epidermis and dermis. Key cellular components include Langerhans, dendritic, T, and B cells, with T cells playing a crucial role in adaptive immunity. These immune cells and molecules are also involved in inflammation [34]. The skin maintains homeostasis through the bidirectional interaction of peripheral neuro–endocrine–immune functions regulated by the CNS, endocrine system, and immune system [35].

**Traumas**

Wound healing is a physiological process involving various cell types interacting in a specific spatial and temporal sequence. It can be divided into four phases: hemostasis, inflammation, proliferation, and maturation [36]. If skin wounds are not successfully repaired on time, they can become “chronic ulcers”, often associated with diabetes, obesity, cardiovascular diseases, and other medical conditions [37]. The impaired expression and regulation of nerve growth factors, reduced cutaneous nerve density, and decreased epidermal innervation contribute to delayed healing [38,39]. Neuropathy, resulting in the loss of protective sensation, and neuropeptide deficiency, leading to reduced trophic action, can cause trauma, increased pressure on the foot’s skin, and a diminished tissue injury response, potentially turning acute wounds into chronic ones and hindering healing [39]. Minor fiber dysfunction is an early feature in patients with type II diabetes mellitus, involving functional and organic abnormalities in unmyelinated C fibers. This may be clinically silent or manifest as a burning sensation in the foot and neurovascular abnormalities linked with impaired blood and immunostained nerves in the skin. Functional neurovascular unit dysfunction, with reduced neuropeptide levels in the blood, can lead to insulin resistance, triggering an inflammatory response and slowing wound healing [40].

Nerve repair occurs primarily through axonal growth during the early stages of trauma and scarring, much slower than granulation tissue formation. Sensory or motor nerve injuries can delay and hinder cutaneous wound healing in the affected area [41]. In a study by Canimdat et al. [42], the hemi-transection of the spinal cord was performed in rats, revealing a significant delay in wound healing in denervated areas compared to normal areas 1 week post-injury. In a study by Kim et al. [43], a linear incision was made in the skin of rats. Disrupting sensory afferents had no significant effect on wound healing, while disrupting sympathetic efferents demonstrated their importance in the healing process. Stein et al. [44] created a denervated skin zone in rats by performing a half-spinal cord dissection. They then created a skin defect symmetrically within the denervated area on the right side of the dorsum and a normal nerve area on the left side. Delayed wound healing, including slower wound contraction and epithelialization, was observed in the denervated skin area, suggesting that sensory deficits negatively affect cutaneous wound healing. In another study by Stelnicki et al. [45], fetal skin wounds with denervated hemi-posterior and incisional wounds were repaired. The study found that the wounds in the denervated group healed completely and without scarring, while wounds in the non-denervated group failed to heal. This suggests that scarless wound healing depends on neuromodulatory factors originating from the CNS.

**Nervous system disorder**

Skin disorders related to the nervous system include conditions such as itch, neurodermatitis, and sensory deficits. Itch, the second most significant form of nociception after pain, is a key symptom in dermatologic disorders with various causes and underlying mechanisms. Itch is classified into different types: peripheral itch, neurogenic itch (caused by damage to afferent nerve fibers), central neurogenic itch (originating in the CNS without nerve damage), and psychogenic itch [46]. Neurogenic itch arises from pathology in afferent pathways, as seen in post-herpetic neuralgia and sensory anomalous spinal pain [47]. Unmyelinated type C and thinly myelinated type Aδ nerve fibers primarily transmit itch signals in the skin. The interaction between the immune and the nervous systems modulates itch transmission in the skin, spinal cord, and brain [48].

Neurodermatitis is a chronic skin condition characterized by recurrent itching, skin thickening, deep dermal furrows, and mossy changes. It commonly affects the posterior cervical region, elbows, wrists, sacral region, and ankles. More prevalent in young adults, particularly females, it is often triggered by dysfunction in the cerebral cortex’s excitatory and inhibitory functions, with psychiatric factors playing a significant role [49]. Patients with neurodermatitis often experience emotional tension, anxiety, and neurasthenia. Itching is typically the initial symptom, and scratching worsens the condition, leading to distress, insomnia, and a vicious cycle of “itching–scratching–itching” that increases the likelihood of recurrent lesions. While the exact pathogenesis is not fully understood, it is generally believed to be closely linked to cortical excitation and abnormal nerve function [50]. Mental stimulation, overwork, insomnia, and scratching are common triggers of neurodermatitis. Depression and anxiety, both triggers and significant accompanying symptoms, highlight the need for appropriate psychoemotional treatments to alleviate psychological stress during disease management [51].

Sensory deficits involve the impaired perception of stimuli, affecting psychological processes and disrupting motor feedback, potentially causing motor dysfunction. The skin has three types of sensory nerve fibers: unmyelinated C-fibers, responsible for injurious sensations, temperature, and slow nociception; myelinated Aδ-fibers, which mediate mechanical stimuli, pressure, temperature, and fast pain; and large myelinated Aβ fibers, which regulate touch and pressure sensations via specialized structures [52]. Sensory deficits manifest as itching, burning, tingling, numbness, reduced sensation, coldness, or pain, often resulting from nerve damage, impingement, or irritation rather than a primary skin condition. Common symptoms of cutaneous sensory disorders include scalp dysesthesia, trigeminal dystrophy syndrome, sensory abnormalities, and brachioradial pruritus [53]. When treated with conventional medium-thickness skin grafts, extensive burns causing the partial or complete destruction of peripheral nerves in the skin often result in poor discriminatory sensitivity, perceptual hypersensitivity, and dysesthesia during healing [54]. Incomplete or disrupted nerve regeneration within grafted tissue is a primary cause of inadequate skin sensation recovery. These sensory deficits often result in functional impairments and a diminished quality of life.

Skin nerve issues may also result from bacterial and viral infections, including leprosy and varicella-zoster. Leprosy is a chronic granulomatous bacterial infection caused by *Mycobacterium leprae* that predominantly affects the skin and peripheral nerves. In leprosy, the abnormal activation of specific B and T lymphocytes can disrupt cellular homeostasis mechanisms. The progression of leprosy is strongly linked to the abnormal activation or apoptosis of lymphocytes. Mycobacterium leprae primarily targets the peripheral nervous system, resulting in diverse clinical manifestations typical of this infection [55]. Skin injuries can impact cutaneous peripheral nerves, such as the posterior tibial, elbow, and medial and lateral peroneal nerves [56], resulting in significant sensorimotor impairments and chronic neurogenic pain. These conditions are a major cause of disability globally [57,58].

The varicella-zoster virus, a neurotropic human herpesvirus, causes chickenpox during primary infection in children and can reactivate later in life as herpes zoster (shingles). It primarily targets cranial, spinal root, and autonomic ganglia along the neuraxis, where it remains dormant for life. Reactivation typically occurs with aging or in response to specific triggers [59]. Herpes zoster commonly affects skin regions innervated by peripheral nerves, particularly those supplied by the trigeminal and spinal nerves [60]. It is uncommon for the condition to involve multiple nerve segments simultaneously, and cases affecting non-adjacent skin segments are exceedingly rare in both immunocompetent and immunocompromised individuals [61]. Shingles can cause nerve damage, with post-herpetic neuralgia being the most common complication. Typically confined to the area affected by the shingles outbreak, this condition can persist for months or longer. The virus is also known to cause permanent nerve damage in some cases [62].

**Endocrine and immune disorders**

Endocrine dysfunction can cause various skin disorders. In hypothyroidism, patients often experience cold, mottled, dry skin and coarse, brittle hair due to reduced sebum production. Additionally, these individuals have a higher prevalence of autoimmune conditions such as herpes-like dermatitis, pemphigus vulgaris, vitiligo, and autoimmune urticaria [63]. Melasma and vitiligo are strongly associated with thyroid autoimmunity. Melasma, characterized by localized facial hyperpigmentation, is influenced by sun exposure, genetic factors, and female hormones. In contrast, vitiligo is an acquired disorder marked by skin depigmentation [64]. Studies have shown changes in the number and distribution of skin nerve fibers in vitiligo patients, including those secreting neuropeptide Y (NPY) and calcitonin gene-related peptide (CGRP), as well as fibers reactive to the low-affinity nerve growth factor receptor (NGFr-IR) [65,66]. Patients with vitiligo show increased levels of NPY and catecholamines, including dopamine, epinephrine, and norepinephrine [67]. These catecholamines can induce vasoconstriction, hypoxia, and excessive ROS production, ultimately resulting in melanocyte death [68].

Skin-immune-dysfunction-related diseases include psoriasis, systemic lupus erythematosus (SLE), eczema, and rosacea. Psoriasis, a chronic inflammatory skin condition with a significant genetic predisposition and autoimmune features [69], is characterized by increased skin nerve fibers. These fibers are densely innervated around immune cells and keratinocytes. Kou et al. [70] found that patients with itchy psoriasis exhibit an increased density of epidermal nerves, heightening the cutaneous neural sensitivity and lowering the threshold for itching, thereby exacerbating pruritus.

The pathogenesis of psoriasis involves plaque formation in genetically predisposed individuals through plasmacytoid dendritic cells and type I interferon activation. This leads to epidermal thickening (echinodermis), the rod-like elongation of rete ridges, and the dilation of dermal papillae blood vessels. Various immune cell subsets, such as IL-17-secreting intrinsic lymphoid cells, Th17 and Tc17 cells, IFN-γ-secreting Th1 and Tc1 cells, neutrophils, activated macrophages, and dendritic cells, infiltrate the skin. Through lymphatic drainage, the initial immune response establishes a self-sustaining inflammatory cycle that perpetuates disease activity. During treatment-induced remission, lysed psoriatic plaques contain tissue-resident memory cells, typically CD8^+^IL-17-positive or CD8^+^IFN-γ-positive cells. Chen et al. [71] demonstrated through RNA-seq analysis that peripheral-sensory-nerve-related genes are disrupted in psoriasis patients and that dermal nerve fibers contribute to disease progression by linking epidermal keratinocytes with immune cells. Neurointervention may offer a promising new approach to psoriasis treatment in the future.

Systemic lupus erythematosus is a chronic, multiorgan autoimmune disease characterized by the production of autoantibodies and antinuclear antibody immune complexes [72]. Patients often present with rash, periungual erythema, and skin ulcers. Some may also experience neuropathy symptoms, including pain, numbness, and burning, typically secondary to small fiber neuropathy [73]. Neuropathy signs include significant damage to fine nerve fibers in the lower and upper extremities and decreased epidermal nerve fiber density [74]. Small fiber neuropathy is a peripheral nerve condition primarily affecting myelinated A-delta fibers and unmyelinated C-fibers [75]. SLE is generally believed to have a genetic basis, with environmental factors such as ultraviolet rays, infections, drugs, and diet disrupting the immune system balance. This leads to increased cellular regulation, reduced apoptotic clearance, abnormal immune cell activation, and the production of autoantibodies, ultimately causing damage to various tissues and organs. Recent studies have identified key pathways in the pathogenesis of SLE, including the cytokine signaling pathway, IFN-α/β signaling pathway, Toll-like receptor signaling pathway, and T and B lymphocyte receptor signaling pathway. These pathways highlight crucial cells and mechanisms involved in SLE and have become important targets for research on targeted therapies [76].

Eczema is a chronic inflammatory condition marked by itching, rashes, and intense irritation. Research has shown an increased association between mast cells and nerves in eczema lesions, with elevated SP and CGRP fiber levels in affected areas. Additionally, there is a significant reduction and damage to dermal nerve fibers, contributing to itching [77].

Rosacea is a common skin disorder that primarily affects the face, and it has historically been classified into four subtypes: erythematous capillary, papulopustular, sarcoidal, and ocular rosacea [78]. The pathophysiology of rosacea is thought to result from a combination of disease processes that contribute to the clinical manifestations in each patient [79,80]. Rosacea is characterized by flushing, transient or persistent erythema, capillary dilation, papules, pustules, abscesses, and (micro)oedema [81]. Patients often report tingling or burning pain, with pruritus being rare. The exact pathogenesis of rosacea remains unclear, but it involves immune dysfunction, neurovascular dysregulation, and stress hormones. Like many other inflammatory skin diseases, rosacea is triggered by external physical, chemical, and biological stimuli that affect the cutaneous nervous and immune systems. Sensory neuron density is increased in rosacea [82], and there is an elevated number of immune cells, such as mast cells, in affected skin [83].

## 3. The Role of Nerves in the Regeneration of Skin Trauma Regeneration and Scar Healing

### 3.1. Nerves May Promote the Healing of Skin Wounds

Sensory and autonomic nerves influence various physiological processes in the skin, including inflammatory responses, immune defense, apoptosis, cell proliferation, and wound healing [84]. Sensory nerves can be classified into four categories: Aα fibers, Aβ fibers, Aδ fibers, and slow-conducting C fibers. These fibers respond to various stimuli, including trauma, heat, cold, osmotic changes, mechanical stimuli, ultraviolet rays, toxins, allergens, and microorganisms. They innervate the epidermis, dermis, and subcutaneous adipose tissue, forming a three-dimensional network [85]. Most nerve fibers are located in the middle and papillary dermis. The epidermis, blood vessels, and skin appendages, such as hair follicles, sebaceous glands, and sweat glands, are innervated by various sensory nerve subtypes. In addition to serving as afferent systems that transmit stimuli from the skin to the central nerves, sensory nerves also function in an efferent neurosecretory manner through their terminals. In contrast to sensory nerve fibers, autonomic nerve fibers comprise only a small fraction of dermal nerve fibers and are primarily located in the dermis. These fibers regulate blood circulation, lymphatic function, and skin appendages. Autonomic nerves mainly release neuropeptides such as acetylcholine (ACh), NPY, CGRP, and vasoactive intestinal peptide (VIP), along with neuromodulators like tyrosine hydroxylase [86]. These neuropeptides play a positive role in wound healing in clinical practice. However, the healing rate is slower in patients with CNS injuries. While skin wounds heal in these patients, chronic ulcers in diabetic patients with peripheral neuropathy are more challenging to treat. In contrast, neuritis due to leprosy does not develop proliferative scarring, suggesting that neuromodulation plays a critical role in the healing process of skin wounds.

Most studies conclude that nerves promote skin wound regeneration and scar healing. Nerves trigger neurogenic inflammatory responses and activate local inflammatory mediators at the injury site [87]. They also enhance blood supply to the wound and surrounding tissues by dilating local blood vessels, promoting DNA synthesis, and stimulating the proliferation of endothelial cells, vascular smooth muscle cells, keratinocytes, and fibroblasts (FBs) [88]. Nerves modulate wound healing by interacting with the neuro-immune response system, releasing neuropeptides, and mobilizing immune cells. They accelerate healing by promoting neovascularization through neurotrophic effects, stimulating the transformation of fibroblasts into myofibroblasts, increasing the expression of collagen types I and III, and enhancing collagen contraction, all contributing to wound healing [89,90].

Romana-Souza B et al. [91] found that the sympathetic neurotransmitter dopamine inhibits skin wound healing through a strong antiangiogenic effect. They also discovered that sensory nerves significantly impact wound healing more than motor nerve fibers, suggesting distinct roles for sympathetic, sensory, and motor nerves in the healing process.

### 3.2. Neuropeptides Promote Wound Healing

As a neuro–immune–endocrine organ, the skin is closely linked to the peripheral sensory nervous system (PNS), autonomic nervous system (ANS), and CNS. It contains numerous neuropeptides produced by skin cells or immune-system-resident cells and released from sensory nerve fibers [92]. Nerves containing and releasing neuropeptides, primarily Aδ and C fibers [93,94], can modulate the inflammatory response through the local release of neuropeptides. These neuropeptides regulate both acute and chronic inflammatory processes in the skin and provide dense innervation to most organs and tissues, particularly blood vessels [95].

**Figure 4:**
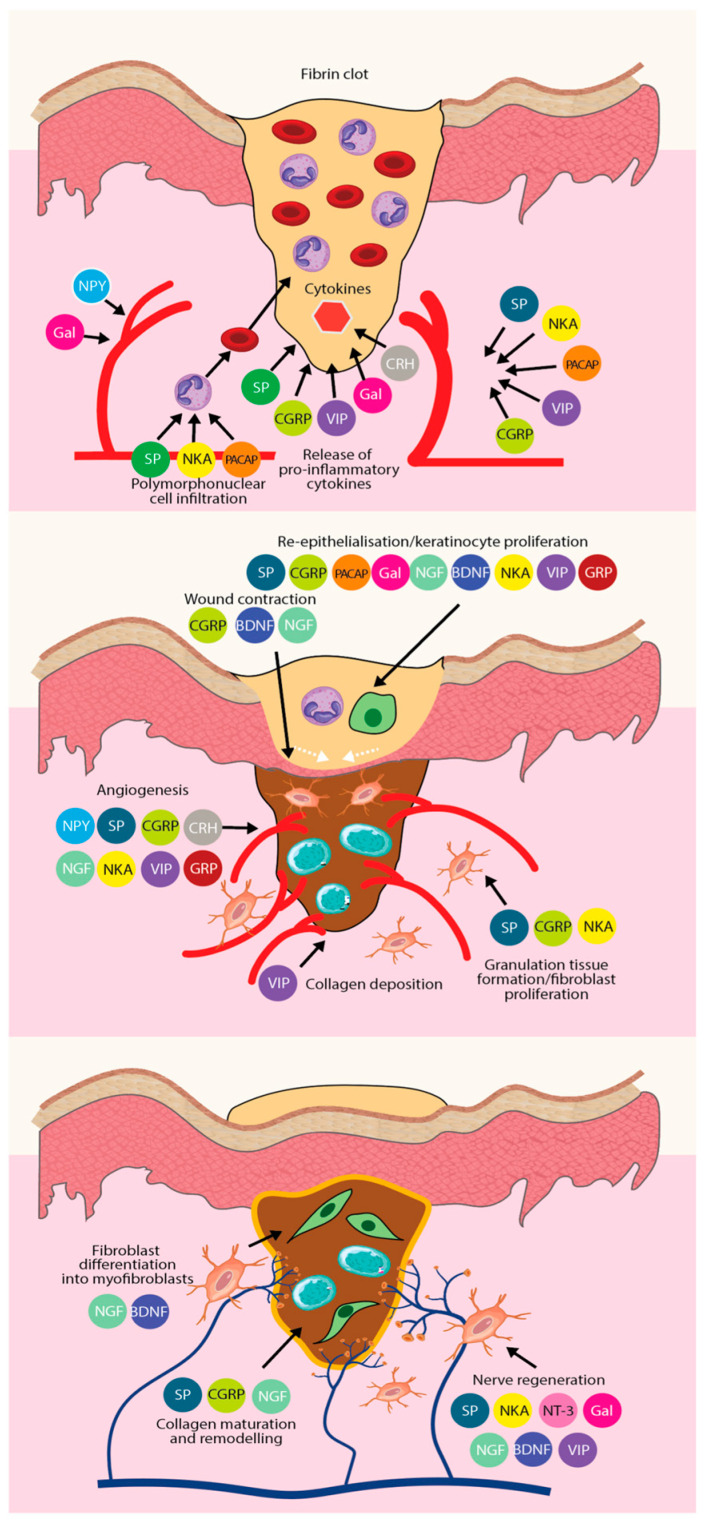
The role of different types of neuropeptides in the three phases of wound healing, and the synergistic effect of each neuropeptide to promote tissue regeneration and wound repair. From top to bottom, there is the inflammatory phase, the proliferative phase, and the remodeling phase.

SP is widely present in the central and peripheral nervous systems and exhibits a strong affinity for neurokinin receptors. It directly enhances re-epithelialization by acting on keratinocytes and promotes dermal fibroblast differentiation, proliferation, and migration. Additionally, SP facilitates the transformation of fibroblasts into myofibroblasts and increases the fibroblast secretion of matrix metalloproteinases through secondary epidermal growth factor expression, aiding collagen degradation and playing a crucial role in granulation tissue remodeling [96]. CGRP, primarily secreted by sensory nerve C-fibers, is the most abundant neuropeptide in the skin. It exhibits potent vasodilatory effects and promotes endothelial cell proliferation and angiogenesis, as demonstrated in in vitro and in vivo studies. CGRP promotes skin wound healing by accelerating local wound contraction, stimulating keratinocyte proliferation, and enhancing NGF release. It works synergistically with SP, as SP induces CGRP release, and CGRP can further amplify SP activity. VIP promotes nerve regeneration, granulation tissue growth, and angiogenesis. PACAP, a key vasoregulatory factor in the skin, supports keratinocyte proliferation. Glycopeptide (Gal) is widely distributed in the central and peripheral nervous systems, and it binds to sites in blood vessels, sweat glands, and keratinocytes, contributing to skin function. Gastrin-releasing peptide (GRP) enhances keratinocyte proliferation and migration while promoting neoangiogenesis, playing a critical role in skin repair by regulating healing markers. Neuropeptide Y, extensively distributed in the central and peripheral nervous systems, contributes to skin function and repair processes. Angiogenic factors serve as downstream targets of NPY, which directly stimulates endothelial cell proliferation and migration, promoting neovascularization. Additionally, platelet lysates produce NPY, enhancing endothelial cell proliferation in a calcium-dependent manner and inducing angiogenesis [97] (Figure 4).

## 4. The Role of Exosomes in the Regulation of Cutaneous Nerve Injury

Growing evidence demonstrates that exosomes derived from various stem cell sources effectively promote wound healing, tissue repair, and regeneration and aid in diagnosing and treating diseases. These advancements offer a promising approach for treating cutaneous nerve injuries caused by diverse pathologies, paving the way for innovative cell-free therapies.

### 4.1. Regulatory Role of Exosomes in Skin Damage

Exosomes derived from mesenchymal stem cells (MSCs) contain various bioactive substances that significantly contribute to wound healing. By delivering active proteins and nucleic acids to target cells, these exosomes influence key aspects of the healing process, including the inflammatory response, cell proliferation, tissue remodeling, angiogenesis, and matrix reconstruction. This accelerates wound healing while inhibiting the formation of keloid scars [98,99].

For example, umbilical cord blood MSC-derived exosomes (UMSC-Exos) have been shown to accelerate wound healing and skin regeneration. These exosomes promote fibroblast aggregation and stimulate the secretion of NGF, which supports nerve regeneration in the skin. Research by Zhu et al. [100] demonstrated that UMSC-Exos enhanced the growth and migration of dermal fibroblasts, promoting fibroblast aggregation and significantly improving skin nerve regeneration in vivo. This highlights the crucial role of UMSC-Exos in cutaneous nerve repair, wound healing, and skin regeneration.

Similarly, exosomes derived from gingival mesenchymal stem cells (GMSC-Exos) possess favorable biological properties. They are applied in tissue engineering and various fields, including bone defect treatment, wound healing, periodontal tissue regeneration, tendon regeneration, and peri-implantitis [101]. Shi et al. [102] found that combining GMSC-Exos with a chitosan/sericin gel sponge resulted in higher wound healing rates, increased re-epithelialization, elevated collagen content, enhanced microvessel density, and a greater presence of nerve fibers compared to other groups. GMSC-Exos effectively promoted wound repair, and loading exosomes onto porous chitosan/silk-protein gel sponges proved to be an effective method for applying exosomes in wound healing. Furthermore, the chitosan/silk-protein gel sponge positively affected wound repair. However, research on the impact of exosomes on nerve fiber regeneration in skin wound healing remains limited.

Exosomes derived from MSCs, such as UMSC-Exos and GMSC-Exos, show significant potential in promoting wound healing, skin regeneration, and nerve fiber regeneration in skin wound repair. However, challenges like a low exosome yield and incomplete functionality remain. Further research is needed to clarify the mechanisms behind these effects and explore their broader therapeutic applications.

### 4.2. Regulation of Exosomes in Peripheral Nerve Injury

The repair process of peripheral nerve injury (PNI) is complex, involving changes such as altered Schwann cell phenotypes, macrophage activation, and vascular network reconstruction [103]. Despite advancements in synthetic nerve conduits and surgical techniques, regeneration remains suboptimal. The efficacy of MSC-based therapeutic strategies for PNI is largely due to their paracrine secretion. Exosomes, secreted by cells and acting as key regulatory mediators, have emerged as a novel therapeutic tool for PNI. Studies have shown that transplanted MSCs can differentiate into Schwann cells in vivo, which are crucial glial cells in the peripheral nervous system that facilitate axonal regeneration and growth [104]. Stem-cell-derived exosomes can be internalized explicitly by axons, significantly promoting axon regeneration in vitro and after sciatic nerve injury in vivo [105]. Studies have shown that exosomes from various MSC sources, including menstrual MSCs, bone marrow MSCs (BMSCs), and adipose MSCs, can enhance neuron growth and regeneration [106]. Gingival-derived mesenchymal-stem-cell-derived exosomes have been shown to repair Schwann cells, activate the c-jun innervation phenotype, and upregulate genes associated with Schwann cell dedifferentiation and repair, thereby promoting peripheral nerve regeneration [107,108]. MSC-derived exosomes mediate intercellular communication, deliver genetic material and neurotrophic factors, and regulate axon regeneration in the peripheral nerve microenvironment, facilitating the recovery of PNI. These effects have significant clinical implications for MSC-derived exosome applications [109]. The local injection of GMSC-derived exosomes significantly promotes the regeneration and functional recovery of sciatic nerve axons in mice with crush injuries. GMSCs, GMSC-derived neural progenitor cells (NPCs), or GMSC-derived exosomes may facilitate peripheral nerve regeneration by reprogramming cells into a reparative phenotype characterized by the increased expression of the key transcription factor c-jun and enhanced proliferation [110,111]. Furthermore, GMSC-derived exosomes promoted cell proliferation and axon growth in vitro, resulting in significant nerve fiber regeneration and accelerated wound repair.

Stem-cell-derived exosomes regulate intercellular transport and the expression of miRNAs to promote axon growth. Exosome-mediated miRNA exchange plays a key role in neurological repair. Didiot et al. [112] applied the gene editing of miRNAs in exosomes to study Huntington’s disease. They found that exosomes carrying modified small interfering RNAs could be internalized by host neurons, leading to the differential expression of miRNAs and proteins. After peripheral nerve injury, neuronal cytosol degeneration occurs, interrupting the supply of target-derived growth factors. This disruption prevents the rapid activation of growth factor signaling pathways in neuronal and Schwann cells, hindering nerve regeneration. Studies have shown that exogenous nerve growth factor supplementation in damaged nerve tissues increases the nerve growth factor content in the stump and promotes circulatory homeostasis, aiding nerve tissue regeneration and functional recovery [113]. Adipose-MSC-derived exosomes containing neurotrophic factors, such as fibroblast growth factor 1, brain-derived neurotrophic factor, insulin-like growth factor 1, and nerve growth factor, can be transferred to Schwann cells, promoting axonal regeneration and myelin formation [114]. In addition to carrying growth factors, stem-cell-derived exosomes can activate injured neuronal cells to secrete various active growth factors, including hepatocyte growth factor, insulin-like growth factor 1, nerve growth factor, and stromal-derived growth factor 1. This process involves signaling pathway molecules such as Akt, ERK, and STAT3, revealing how stem-cell-derived exosomes mediate the promotion of neural tissue regeneration [115]. Growth factors in stem-cell-derived exosomes and the activation of secretion signaling pathways offer the potential for regulating atrophic nerve survival and axon growth. Increasing the bioactive molecules in exosomes, potentially through genetic engineering to regulate the expression of nerve growth factor target genes, could enhance the repair of peripheral nerve injuries.

### 4.3. Exosome Modulation in Nerve Injury Pain

In psoriasis, dermal dendritic cells induce the production of IL-17 and IFN-γ by activated T cells. In contrast, they do not produce IL-17 or IFN-γ in normal skin. Studies show that dendritic cells are a primary cell type infiltrating the dermis of psoriasis-affected skin, contributing to the pathogenesis of the disease by releasing inflammatory and chemotactic factors that drive Th1/Th17 cell polarization, among other effects. The interaction between dendritic cells and abnormally activated keratinocytes also contributes to the progression of psoriasis [96]. Recent research has highlighted the role of exosomes, which carry substances such as DNA, RNA, and proteins and are present in various body fluids, in psoriasis [97,98]. Studies have found that tonsil-derived MSCs inhibited the proliferation of bone-marrow-derived dendritic cells (BM-DCs) and suppressed the upregulation of CD86, CD80, and MHC class II molecules induced by lipopolysaccharides (LPSs). This resulted in CD4+ T cells with weaker proliferative and differentiation abilities. Dendritic cells are crucial in inflammation regulation and self-tolerance. Co-culturing MSCs with dendritic cells significantly reduces the chemotactic responsiveness and phenotypic characteristics of mature dendritic cells, offering a potential therapeutic approach for psoriasis [100].

The neuroimmune response triggered by neuronal injury is pivotal in neuropathic pain development [116]. Yu et al. [117] found that MSC-Exo delivery significantly increased the axon branch number and length, with microRNA 133b identified as a key factor in promoting neuronal recovery. Neuropathic pain from nerve injury is challenging to manage, and it often severely affects quality of life. Shiue et al. [118] demonstrated that UCMC-Exos inhibited the glial activation and neuroinflammation induced by spinal nerve ligation (SNL) in rats. Notably, UCMC-Exos provided rapid analgesic effects comparable to conventional pain relief methods. The analgesic effects of these exosomes may involve direct or indirect interactions with neurons and glial cells. Brain-derived neurotrophic factor (BDNF) and glial-cell-derived neurotrophic factor (GDNF), key members of the neurotrophic factor family, support neuronal differentiation, survival, axonal regeneration, and excitability [119]. GDNF protects neuronal cells, and exosome treatment enhances BDNF and GDNF expression in SNL rats, indicating that UCMC-Exos may have neurotrophic properties [120]. With its homing ability and regenerative potential, UCMC-Exo is a promising candidate for treating nerve-injury-induced pain.

Stem-cell-derived exosomes facilitate intercellular communication between nerve cells and other cells within the skin niche. These exosomes are absorbed by neighboring fibroblasts, keratinocytes, and immune cells, influencing their behavior and function. Their cargo modulates cellular processes such as proliferation, migration, and immune responses, enhancing therapeutic outcomes.

### 4.4. Synergistic Modulation of Exosomes in the Treatment of Skin Lesions and Nerve Damage

Diabetic foot ulcers are typical neuropathic ulcers whose pathogenesis is related to peripheral neuropathy and vascular insufficiency in diabetic patients. Sensory neuropathy in diabetic patients causes abnormal plantar pressures, leading to ulcer development. The complex wound microenvironment in diabetic patients is characterized by hyperglycemia, ischemia, hypoxia, and persistent infection, which cause ulcers to repeatedly remain in the inflammatory phase and impede wound healing [121]. Exosomes are secreted by cells with biological properties such as a low immunogenicity, biocompatibility, and long circulation time, making them excellent therapeutic carriers [122,123]. The surface of exosomes is enriched with bioactive components, which enhances the modifying and targeting properties of exosomes [124]. Qiao et al. have shown in their study that exosomes of a specific origin can be used for the treatment of diabetic foot ulcers by modulating pathogenesis-related signaling pathways and cellular functions [125]. MSC-Exos, as the main paracrine component of MSCs, contain a number of neuroprotective and anti-inflammatory small molecules and proteins, Fan et al. proposed that specific miRNAs inherited by exosomes from parental cells, such as let-7a, miR-23a, and miR-125b, have a positive therapeutic effect on alleviating neuroinflammation by targeting the Toll-like receptor 4/nuclear factor-κB signaling pathway [126]. Exosomes promote peripheral nerve regeneration and angiogenesis, can inhibit long-term inflammation, and promote wound re-epithelialization [127,128].

Complex regional pain syndrome (CRPS) is triggered by abnormalities in sympathetic and sensory nerve interactions, resulting in localized skin erythema, atrophy, and ulceration, usually after fracture or trauma [129,130]. Heidrun H Krämer et al. examined skin and serum levels of TNF-α in patients with acute traumatic fractures and in patients with CRPS by sampling patients with acute traumatic fractures. TNF-α expression is locally elevated, and post-traumatic TNF-α signaling contributes to the development of CRPS [131]. Patients with CRPS develop an imbalance in the expression of circulating miRNAs, which act as micro-regulators of gene expression, affecting almost all aspects of cellular processes [132,133,134]. Exosomes reduce pro-inflammatory factors such as TNF-α and IL-1β by inhibiting the NF-κB pathway, as well as repair synaptic plasticity and alleviate neuroinflammation by activating the BDNF/TrkB pathway via miR-21-5p, which also promotes skin ulcer repair while treating CRPS [135,136]. The release and uptake of exosomes play an important role in cellular communication, and the transport of small non-coding RNAs via exosomes regulates gene expression, alleviates pain hypersensitivity, and promotes skin regeneration in CRPS patients [133,134].

Chronic lichen simplex is a chronic pruritic dermatosis characterized by intractable itching that leads to scratching and rubbing, resulting in a vicious cycle of itching–scratching–itching that eventually leads to skin thickening, lichenification, and scaling [137]. Pruritus can be broadly classified into histaminergic itch and non-histaminergic itch. Histamine released by mast cells is the central source of histaminergic itch, and IL-33 further enhances histaminergic itch by activating mast cells and inducing IL-13 secretion [138]. Non-histaminergic itch is primarily associated with G-protein-coupled receptors (GPCRs) and transient receptor potential channels, where signals from the GPCR superfamily converge on the transient receptor potential channel family (TRPs), leading to channel sensitization and activation, which amplifies itch and neuroinflammation [139]. Exosomes reduce the release of the neuropeptide substance P by inhibiting mast cell degranulation and the IL-4/IL-13 pathway, alleviating itch and erythema while repairing the skin barrier [140,141]. At the same time, exosomes can also play a therapeutic role by inhibiting non-histaminergic-itch-associated neuroreceptors and ion channels. Exosomes target TRP, protease-activated receptor 2 (PAR2), and G-protein-coupled receptors (e.g., GPR35) to block dorsal root ganglion (DRG) neuron responses to non-histaminergic pruritogens (e.g., trypticin, IL-31) [142,143]. Exosomes can simultaneously intervene in multiple pathological aspects of itch and treat chronic lichen simplex moss through multi-target, multi-pathway synergistic action.

## 5. MSC-Exo Applications and Challenges

The results of this study highlight the significant potential of MSC-Exo in promoting cutaneous nerve regeneration. In the future, MSC-Exo-based therapy could become a viable strategy for treating cutaneous nerve injuries. However, several challenges remain to be addressed.

First, a large-scale and consistent culture of stem-cell-derived exosomes is necessary. As studies on exosomes increase, standardizing the production process becomes essential. This includes improving uniform production, isolation, characterization, and storage methods and establishing recognized systems for evaluating safety and efficacy. Clinical dosing guidelines must also address seven key aspects: exosomal cell selection and characterization, large-scale production and isolation, purification, component analysis, quality control, storage stability, and product safety.

Transforming exosomes from various cell sources into safe and effective clinical therapeutic products requires strict control measures. These include parental cell selection, the evaluation of immunological and oncogenic effects, and addressing viral contamination risks. The continuous monitoring of and enhancement in existing assessment methods are essential in order to ensure the safety and efficacy of engineered exosomes. The further exploration of optimal exosome sources is necessary due to exosomal heterogeneity. Milk-derived exosomes show potential as an alternative for obtaining large quantities of biocompatible exosomes. However, technologies for their large-scale isolation and purification from compounded milk need refinement [144].

Compared to synthetic carriers, the virus-like size and complexity of exosomes pose challenges for their comprehensive characterization and quality assurance in engineered exosomes [145]. Additionally, the absence of efficient, high-yield methods for isolating and purifying exosomes remains a significant barrier to their large-scale clinical application. Although commercial kits using immunoaffinity membrane adsorption, co-precipitation, and other technologies aim to overcome the limitations of traditional extraction methods, the exosomes obtained often have a low purity and limited visibility under a transmission electron microscope. The industrial production of engineered exosomes is essential for clinical translation, but current purification methods do not meet industrialization standards. Therefore, separation and purification technology remain a significant challenge for large-scale experiments and clinical applications.

Additionally, the storage of exosomes presents a significant challenge. Freeze-drying could be a promising method for commercializing exosome-based therapeutics, but further clinical data are needed. Continued efforts are essential for the clinical translation of exosomes.

Overall, the large-scale application of exosomes requires improvements in production, separation, characterization, storage technologies, and establishing a recognized safety and efficacy evaluation system. Addressing these challenges is essential for advancing their clinical use.

## 6. Conclusions

MSC-Exo-based therapeutic strategies offer a novel approach, with recent advancements highlighting their exceptional potential in treating cutaneous nerve injuries. Stem cell exosome therapy has been demonstrated to significantly promote wound healing in diabetic foot ulcers (DFUs). The therapeutic efficacy of stem cell exosome therapy is attributed to the presence of specific microRNAs (miRNAs) within the exosome that modulate various biological processes, including angiogenesis, inflammatory responses, and cell proliferation and migration. Notably, the therapeutic effect of stem cell exosome therapy is particularly pronounced when the exosome is derived from adipose-derived stem cells (ADSCs) or mesenchymal stem cells (MSCs). Exosomes derived from adipose-derived stem cells (ADSCs) have been shown to enhance endothelial cell function and promote accelerated angiogenesis through the action of microRNA-125b [146]. In addition, the over-expression of microRNA-1248 in ADSCs has been observed to enhance diabetic wound healing by regulating endothelial cell function [147]. The MSC Exosome has been demonstrated to inhibit chronic inflammation by delivering microRNAs (miRNAs), such as miR-146a, which regulate IL-1β expression, alleviate high-glucose-induced inflammatory responses, and improve wound healing [148]. The MSC Exosome has been shown to directly promote endothelial cell proliferation in a high-glucose environment, migration, and angiogenic capacity. Furthermore, the MSC Exosome has been demonstrated to improve extracellular matrix remodeling through the regulation of fibroblast–macrophage interactions, thereby aiding wound healing [146].

Extracellular vesicles derived from bone marrow MSCs have been shown to attenuate the inflammatory response of CRPS by inhibiting pro-inflammatory factors (e.g., TNF-α and IL-6). Preliminary clinical studies have shown that these extracellular vesicles can safely alleviate pain symptoms [147]. Furthermore, exosome-borne microRNAs (miRNAs) have been shown to potentially mitigate central sensitization and autonomic dysfunction by modulating neurotransmitter release or neuron–glia interactions [148].

As engineered exosomes evolve, their application expands beyond MSC-Exo-based therapy. Various exosomal drug delivery systems have been developed to enhance therapeutic efficacy through synergistic effects. These promising systems can transport gene therapies, chemical drugs, herbal ingredients, and more. Compared to synthetic drug delivery systems like liposomes, nanoparticles, microspheres, and microemulsions, exosomal drug delivery systems offer advantages such as a low immunogenicity, high tissue permeability, and similarity to cell membranes. However, a significant gap remains between laboratory research and the clinical industrialization of exosome-based drug delivery systems. Successfully transitioning laboratory findings into industrial-scale production remains a challenge. However, with the advancements in engineered exosome technology and drug delivery mechanisms, exosome-based drug delivery systems are expected to see widespread clinical application.

## Data Availability

The original contributions presented in the study are included in the article; further inquiries can be directed to the corresponding authors.
